# AI-accelerated 3D gradient echo versus ultrashort echo time MRI for lung nodule detection and measurement

**DOI:** 10.1093/radadv/umag029

**Published:** 2026-07-07

**Authors:** Alexander W Marka, Kilian Weiss, Hannah Rosenkranz, Bernadette Scherer, Lisa Adams, Keno Bressem, Nicolas Lenhart, Tristan Lemke, Florian T Gassert, Marcus R Makowski, Daniela Pfeiffer, Gregor S Zimmermann, Seyer Safi, Dimitrios C Karampinos, Markus Graf, Sebastian Ziegelmayer

**Affiliations:** Institute for Diagnostic and Interventional Radiology, School of Medicine and Health, TUM Klinikum, Technical University of Munich (TUM), Munich 81675, Germany; Philips GmbH Market DACH, Hamburg 22335, Germany; Institute for Diagnostic and Interventional Radiology, School of Medicine and Health, TUM Klinikum, Technical University of Munich (TUM), Munich 81675, Germany; Institute for Diagnostic and Interventional Radiology, School of Medicine and Health, TUM Klinikum, Technical University of Munich (TUM), Munich 81675, Germany; Institute for Diagnostic and Interventional Radiology, School of Medicine and Health, TUM Klinikum, Technical University of Munich (TUM), Munich 81675, Germany; Institute for Diagnostic and Interventional Radiology, School of Medicine and Health, TUM Klinikum, Technical University of Munich (TUM), Munich 81675, Germany; Institute for Diagnostic and Interventional Radiology, School of Medicine and Health, TUM Klinikum, Technical University of Munich (TUM), Munich 81675, Germany; Institute for Diagnostic and Interventional Radiology, School of Medicine and Health, TUM Klinikum, Technical University of Munich (TUM), Munich 81675, Germany; Institute for Diagnostic and Interventional Radiology, School of Medicine and Health, TUM Klinikum, Technical University of Munich (TUM), Munich 81675, Germany; Institute for Diagnostic and Interventional Radiology, School of Medicine and Health, TUM Klinikum, Technical University of Munich (TUM), Munich 81675, Germany; Institute for Diagnostic and Interventional Radiology, School of Medicine and Health, TUM Klinikum, Technical University of Munich (TUM), Munich 81675, Germany; Division of Respiratory Medicine, Department of Internal Medicine I, School of Medicine and Health, Klinikum rechts der Isar, TUM University Hospital, Technical University of Munich, Munich 81675, Germany; Division of Thoracic Surgery, Department of Surgery, School of Medicine and Health, TUM Klinikum, Technical University of Munich (TUM), Munich 81675, Germany; Institute for Diagnostic and Interventional Radiology, School of Medicine and Health, TUM Klinikum, Technical University of Munich (TUM), Munich 81675, Germany; Institute for Diagnostic and Interventional Radiology, School of Medicine and Health, TUM Klinikum, Technical University of Munich (TUM), Munich 81675, Germany; Institute for Diagnostic and Interventional Radiology, School of Medicine and Health, TUM Klinikum, Technical University of Munich (TUM), Munich 81675, Germany

**Keywords:** lungs, nodules, MRI, low-dose CT, CSAI, UTE

## Abstract

**Background:**

Ultrashort echo time (UTE) sequences are widely used in pulmonary MRI due to favorable lung parenchyma signal but are limited by acquisition time and complex reconstruction.

**Purpose:**

To compare an AI-enhanced 3D gradient echo sequence with a UTE sequence for lung nodule detection and Lung-RADS classification, using CT as the reference standard.

**Materials and Methods:**

In this single-center observational study, patients with at least one CT-detected lung nodule underwent pulmonary MRI. MRI included a respiratory-gated AI-accelerated 3D gradient-echo sequence (CSAI-GRE) and a respiratory-gated 3D UTE sequence, both acquired with comparable scan times. Three radiologists independently assessed image quality, nodule detection, and Lung-RADS classification. CT served as the reference standard. Statistical analyses included paired tests for image quality, mixed-effects modeling for lesion-level detection, and *κ* statistics for Lung-RADS agreement.

**Results:**

This study included 54 patients (mean age, 65 ± 12 years; 23 women [43%]) with 97 pulmonary nodules. Subjective image quality was higher for CSAI-GRE than UTE (mean score, 4.30 vs 3.91; *P* = .005). Lesion-level detection was 96.9% for CSAI-GRE and 92.8% for UTE; mixed-effects analysis showed higher conditional detection odds for CSAI-GRE (OR, 10.2; 95% CI, 2.4-43.3; *P* = .002). MRI-CT agreement for Lung-RADS categorization ranged from substantial to almost perfect across readers (*κ* range, 0.79-0.93 for CSAI-GRE; 0.72-0.94 for UTE). Lung-RADS reclassification relative to CT occurred in 12.3% (20/162; 95% CI, 8.1-18.3) of CSAI-GRE and 20.8% (33/159; 95% CI, 15.2-27.7) of UTE reader assessments, including 5.6% (9/162; 95% CI, 3.0%-10.2%) and 10.7% (17/159; 95% CI, 6.8%-16.5%) management-relevant changes, respectively.

**Conclusion:**

CSAI-GRE and UTE enabled reliable pulmonary nodule detection. CSAI-GRE provided superior subjective image quality and slightly higher detection performance than UTE. However, the Lung-RADS reclassification rates suggest that MRI-based Lung-RADS categorization may not yet be sufficiently robust for routine clinical application.

**Abbreviations** 

AI = artificial intelligence, CSAI = reconstruction algorithm combining parallel imaging, compressed sensing, and deep learning, GRE = 3D gradient recall echo, ICC = intraclass correlation coefficient, Lung-RADS = Lung Imaging Reporting and Data System, UTE = ultrashort echo time

**Summary** 

An artificial intelligence-enhanced 3D gradient echo MRI sequence demonstrated higher image quality and comparable lung nodule measurement and classification compared with ultrashort echo time sequence.

**Key Results** 

- AI-accelerated 3D gradient echo sequence (CSAI-GRE) achieved a 96.9% nodule detection rate compared with 92.8% for UTE (*P* = .002).
- CSAI-GRE demonstrated higher subjective image quality ratings (mean score, 4.30 vs 3.91; *P* = .005) compared with UTE.
- Lung-RADS reclassification relative to CT occurred in 12.3% (20/162; 95% CI, 8.1-18.3) of CSAI-GRE and 20.8% (33/159; 95% CI, 15.2-27.7) of UTE assessments, including management-relevant changes in 5.6% (9/162; 95% CI, 3.0%-10.2%) and 10.7% (17/159; 95% CI, 6.8%-16.5%), respectively.

## Introduction

Advances in pulmonary MRI techniques have substantially broadened their clinical utility.^1^ Ultrashort echo time (UTE) imaging enables visualization of the lung parenchyma by detecting short T2* signals^2–4^ and has demonstrated feasibility in pulmonary nodule detection.^5–8^ However, UTE sequences rely on non-Cartesian sampling and complex reconstruction strategies, which may limit workflow efficiency and broader clinical implementation.^9^^,^^10^ Although recent trajectory refinements such as FLORET (fermat looped, orthogonally encoded trajectories) have improved sampling efficiency^11^, Cartesian acquisitions benefit from widely available reconstruction frameworks combining parallel imaging, compressed sensing, and deep learning. These approaches enable a highly accelerated 3D gradient echo sequence with Cartesian sampling.^6^^,^^8^

Although both UTE and accelerated Cartesian gradient echo techniques have shown promise for pulmonary nodule detection, direct comparisons under comparable motion management and acquisition times remain limited. In particular, it is unclear whether AI-accelerated Cartesian gradient echo imaging can achieve detection performance, measurement agreement, and Lung CT Screening Reporting and Data System (Lung-RADS) classification comparable to or exceeding that of 3D UTE.

Therefore, the purpose of this study was to compare a respiratory-gated 3D UTE sequence with a respiratory-gated AI-enhanced 3D gradient echo sequence for pulmonary nodule detection, measurement accuracy, and Lung-RADS categorization, using chest CT as the reference standard.

## Materials and methods

### Patients

**Figure 1:**
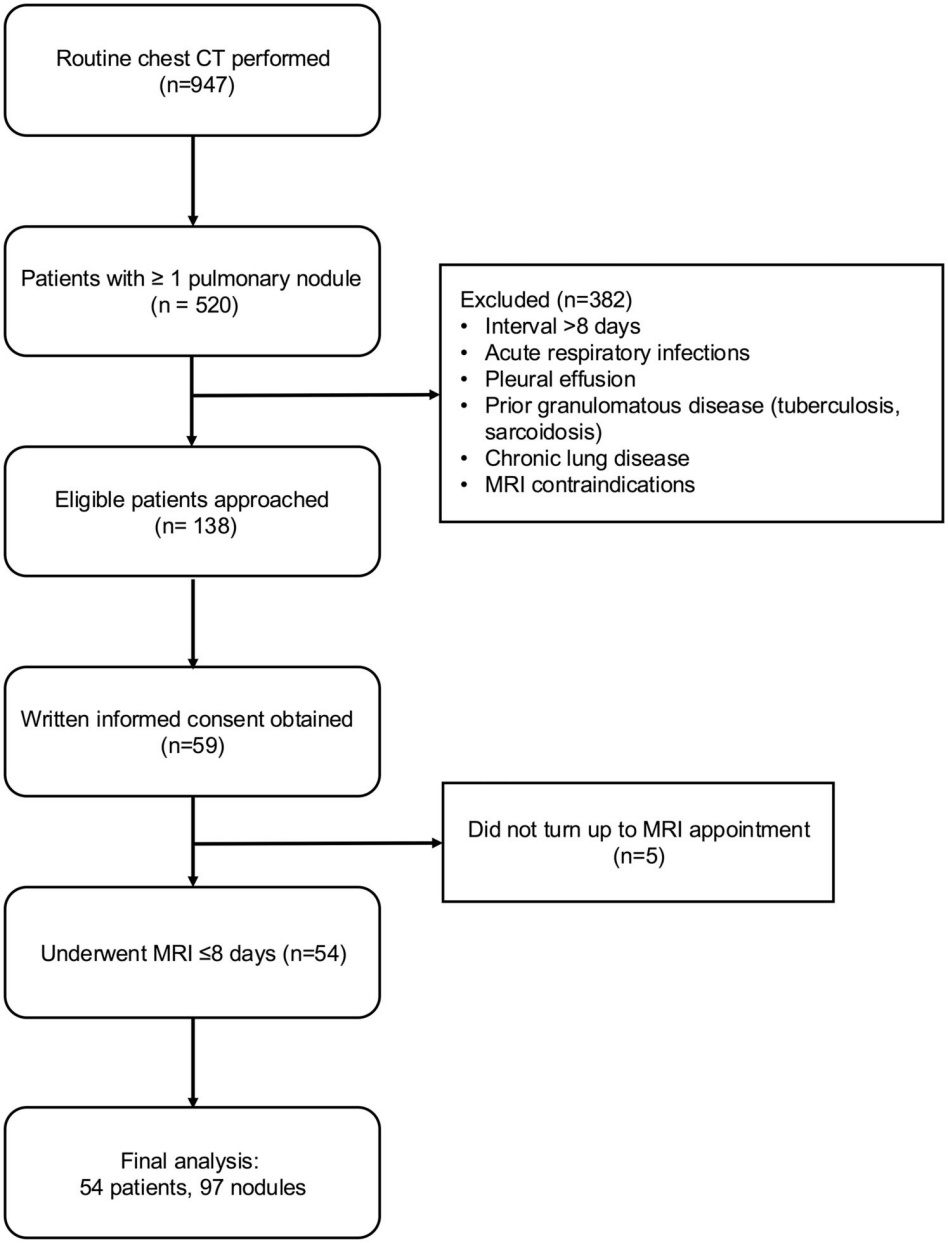
STARD cohort diagram.

This single-center study was approved by the local ethical review board (reference number: 2025-34-S-SB). Chest CT examinations were performed as part of routine clinical care between February and June 2025 (CT indications summarized in Table 1). Written informed consent was obtained from all participants. Patients with at least one lung nodule on clinically indicated chest CT were contacted and asked to undergo 2 additional MRI sequences as part of the research protocol; those undergoing MRI within 8 days were enrolled. Exclusion criteria included nodules without sufficient clinical or imaging follow-up to permit reference-standard lesion classification (eg, benign, metastatic, or primary lung malignancy), advanced chronic lung conditions likely to impair nodule detection (eg, severe emphysema, advanced pulmonary fibrosis, extensive bronchiectasis), acute respiratory infections (including pneumonia), prior granulomatous disease (eg, tuberculosis or sarcoidosis), pleural effusion, age <18 years, pregnancy, and standard contra indications to MRI. Atelectasis was excluded only if it substantially obscured nodule evaluation. Only pulmonary nodules >3 mm on CT were included; no maximum nodule size criterion was applied. All primary lung cancers were confirmed histologically. Benign nodules were defined by typical imaging features and/or longitudinal stability. The patient selection process is illustrated in Figure 1.

**Table 1:** Patient characteristics.

Characteristic	Value
Sex	
Men	31/54 (57.4%)
Women	23/54 (42.6%)
Age (y)	65 ± 12 [35-82]
Men	65 ± 12 [35-82]
Women	66 ± 10 [39-80]
Time between CT and MRI (days)	4 ± 4 [0-8]
CT indication	
Oncologic staging/follow-up	31 (57.4%)
Infection/inflammatory evaluation	10 (18.5%)
Unexplained chest pain	9 (16.6%)
Pulmonary embolism	4 (7.4%)
Lesion etiology	
Metastasis	49 (50.5%)
Primary lung cancer	21 (21.6%)
Inflammatory	14 (14.4%)
Granuloma	13 (13.4%)

Data are presented as *n* (%), unless otherwise indicated. Continuous variables are presented as mean ± SD [range]. Percentages are calculated based on the total number of patients (*n* = 54) or nodules (*n* = 97), as appropriate.

### CT image acquisition

Thin-section chest CT examinations were performed on a 320-detector row system (IQon—Spectral CT, Philips Healthcare, Best, The Netherlands). Scans were acquired during full inspiration using 120 kV and automated tube current modulation, with a mean effective tube current of 59 mAs. Images were reconstructed in axial plane with a dedicated lung kernel (YA), 1-mm slice thickness, 1-mm slice interval, and iterative reconstruction (iDose level 6). All CT examinations were contrast-enhanced and clinically indicated. Additional acquisition, reconstruction, and contrast media details are provided in Supplementary Material S1.

### MR image acquisition

MRI was performed on a 3 T system (Ingenia Elition X; Philips Healthcare) using anterior and posterior coils.

#### CSAI-3D gradient recalled echo image acquisition

Respiratory-gated accelerated isotropic 3D gradient echo sequence was acquired using Cartesian variable-density *k*-space sampling. Image acceleration (factor 7) was achieved using a reconstruction framework combining parallel imaging, compressed sensing and deep learning (CSAI). The CSAI-GRE sequence represents a *T*1-weighted spoiled gradient-echo acquisition with a very short echo time, resulting in low lung parenchyma signal and increased nodule-to-background contrast. Sequence parameters included repetition time (TR) 2.7 ms, echo time (TE) 0.83 ms, flip angle 10°, field-of-view 320 × 557 × 320 mm^3^, acquired voxel size 1 × 1 × 1 mm^3^ (reconstructed to 0.5 mm isotropic). Estimated acquisition time was ∼3:50 min, depending on the breathing pattern. Reconstruction was based on the Adaptive-CS-Net architecture^12–17^ and implemented as the commercially available SmartSpeed platform (Philips Healthcare, Best, The Netherlands). Further details are in Supplementary Material S2.1.

#### UTE image acquisition

Ultrashort echo time imaging was carried out using the FLORET (fermat looped, orthogonally encoded trajectories) approach, as previously described.^3^^,^^4^^,^^18^^,^^19^ Respiratory-gated 3D acquisitions were obtained with TE 0.095 ms, TR 9.5 ms, flip angle 8°, 3D field-of-view of 350 × 350 × 350 mm^3^, voxel size of 1.5 mm isotropic. Lower spatial resolution than CSAI-GRE was chosen to maintain comparable acquisition times (∼3:30 min). Detailed sequence description and parameters are provided in Supplementary Material S2.2.

### Imaging analysis

#### MRI and CT interpretation

Image evaluation for CSAI-GRE and UTE datasets was performed independently by 2 board-certified radiologists and 1 radiology resident. Readers were blinded to all clinical information and CT findings.

Each reader completed 2 separate sessions. Sequence order was counterbalanced across readers: 2 readers (16-year radiologist and 5-year resident) interpreted UTE datasets first, whereas 1 reader (11-year radiologist) interpreted CSAI-GRE datasets first. A 4-week washout period minimized recall bias.

Images were interpreted on PACS under standard reading room conditions. Readers identified and directly annotated pulmonary nodules >3 mm on the PACS workstation, measured mean axial diameter using electronic calipers, and classified attenuation as solid, part-solid or ground glass. For the 4 part-solid nodules, the maximum diameter of the solid component was measured separately on both MRI sequences. Given the limited number of part-solid nodules, formal agreement analysis for solid-component measurements was not performed. Mean axial diameter was used for statistical analysis and Lung-RADS assignment.

CT examinations were assessed by a board-certified cardiothoracic radiologist who was blinded to MRI findings and did not participate in the MRI assessment. Using the same measurement protocol as for MRI, the radiologist independently assessed the pulmonary nodule and its solid component on CT, which served as the reference standard. After each session, a separate radiology resident collected findings for nodule matching across readers.

#### Lung-RADS and image quality classification

Lung-RADS version 2022^20^ categories were assigned per patient for both MRI sequences. Differences between MRI- and CT-based Lung-RADS categories were recorded as reclassifications. A critical reclassification was defined as a category change altering clinical management according to Lung-RADS recommendations, specifically transitions between surveillance categories (1-4A) and categories recommending more aggressive diagnostic evaluation (4B or 4X) (Supplementary Material S3).^8^ Image quality was assessed independently by each reader using a structured multidimensional 5-point Likert scale (1 = non-diagnostic, 5 = excellent). Assessed domains included overall interpretability, artifact severity, blurring, depiction of anatomical detail, and overall nodule conspicuity. Regional image quality was evaluated using vascular landmarks. Scores ≥4 are considered equivalent to CT and scores ≥3 are regarded as diagnostically sufficient. Detailed scoring definitions are provided in Supplementary Material S4.^6^^,^^21^

### Statistical analysis

All statistical analyses were conducted in R (version 4.2.1, R Foundation for Statistical Computing, http://www.r-project.org; RStudio interface). Patient and nodule characteristics were summarized descriptively. Image quality scores were averaged across readers for each patient and sequence before comparison. Reader-averaged paired scores were compared between CSAI-GRE and UTE using paired Wilcoxon signed-rank tests. Detection performance was assessed at the lesion level. Lesion-level detection was analyzed using generalized linear mixed-effects models with sequence type as the fixed effect and random intercepts for both reader and patient to account for repeated reader assessments and clustering within patients. Because nodules ≥10 mm were detected in all assessments for both sequences, nodule size category was not included as a fixed effect in the primary model. A subgroup mixed-effects analysis was performed for nodules <10 mm. Odds ratios (OR) with 95% CIs are reported as cluster-conditional estimates. Agreement between MRI and CT for nodule attenuation and Lung-RADS classification was assessed separately for each reader using Cohen’s kappa coefficients, whereas inter-reader agreement was evaluated using Fleiss’ kappa statistics. Agreement between MRI and CT measurements of mean nodule diameter was assessed after averaging reader measurements for each nodule and sequence. Agreement was evaluated using the intraclass correlation coefficient (ICC) with a 2-way random-effects model for absolute agreement and by Bland-Altman analysis. CT served as the reference standard. A 2-sided *P-*value of *P*<.05 was considered statistically significant.

## Results

### Participant and nodule characteristics

Overall, 947 patients underwent eligibility screening. Of these, 138 eligible patients were approached, 59 provided written informed consent, and 54 ultimately participated and underwent MRI. Exclusions were due to failure to meet the inclusion criteria (*n* = 382), no written informed consent (*n* = 79), and failure to attend the MRI appointment (*n* = 5). The final cohort comprised 54 patients with 97 nodules (Figure 1).

**Figure 2:**
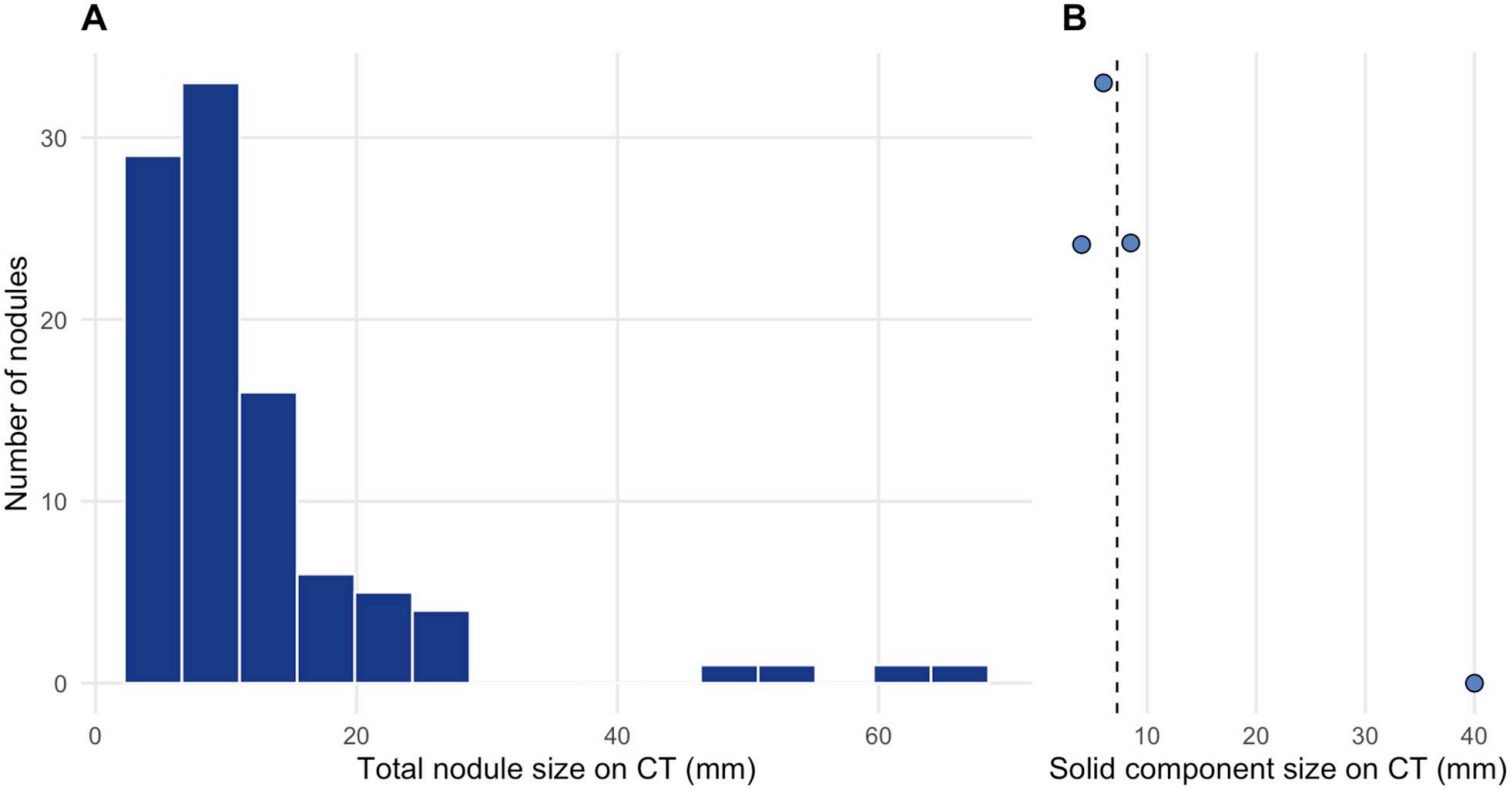
Distribution of CT nodule size and solid component size. (A) Histogram showing the distribution of CT nodule diameters for all nodules included in the study (*n* = 97). The mean CT nodule diameter was 12 ± 11 mm, with a median of 10 mm (interquartile range, 7 mm; range, 4-66 mm). (B) Dot plot showing CT solid component size in part-solid nodules (*n* = 4). The mean solid component size was 15 ± 17 mm, with a median of 7 mm (interquartile range, 11 mm; range, 4-40 mm). Dotted lines indicate median values.

The mean patient age was 65 ± 12 years, and 23 patients (43%) were female. Patient characteristics are summarized in Table 1. Among 97 pulmonary nodules, 50.5% (49/97) represented metastases, 21.6% (21/97) were primary lung cancers, and 27.9% (27/97) were benign. Metastatic nodules were confirmed in 39 cases by follow-up or prior imaging, and in 10 cases by histology. Regarding attenuation, 88 nodules (90.7%) were solid, 4 nodules (4.1%) were part-solid, and 5 (5.2%) were ground-glass nodules. Mean CT nodule diameter was 12 ± 11 mm (range, 4-66 mm). 54 nodules (55.7%) measured <10 mm, with a mean diameter of 7 ± 2 mm (range, 4-9 mm), whereas 43 nodules (44.3%) measured ≥10 mm, with a mean diameter of 19 ± 13 mm (range, 10-66 mm). Nodule characteristics are summarized in Table 2 and Figure 2.

**Table 2:** Nodule characteristics.

Nodule type	Malignant (n = 70)	Benign (n = 27)	Total (n = 97)
Solid			
Number of nodules	65 (92.9%)	23 (85.2%)	88 (90.7%)
CT diameter (mm)	13.3±11.8	7.5±2.4	11.8±10.5
Part-solid			
Number of nodules	4 (5.7%)	0	4 (4.1%)
Total nodule diameter (mm)	19.7±20.7	NA	19.7±20.7
Solid component diameter (mm)	14.6 ± 17.0	NA	14.6 ± 17.0
Pure ground-glass			
Number of nodules	1 (1.4%)	4 (14.8%)	5 (5.2%)
CT diameter (mm)	7.1±NA	18.4±7.8	16.1±8.4

Unless otherwise specified, values are number of nodules with percentages in parentheses. Continuous variables are reported as mean ± SD. Percentages in the Malignant and Benign columns are calculated within each column (ie, out of *n* = 70 and *n* = 27, respectively); percentages in the Total column are calculated out of *n* = 97. Solid component diameter is reported only for part-solid nodules and measured on CT; “NA” indicates not applicable (no solid component/no variability for SD).

### Imaging analysis

#### Image quality

**Figure 3:**
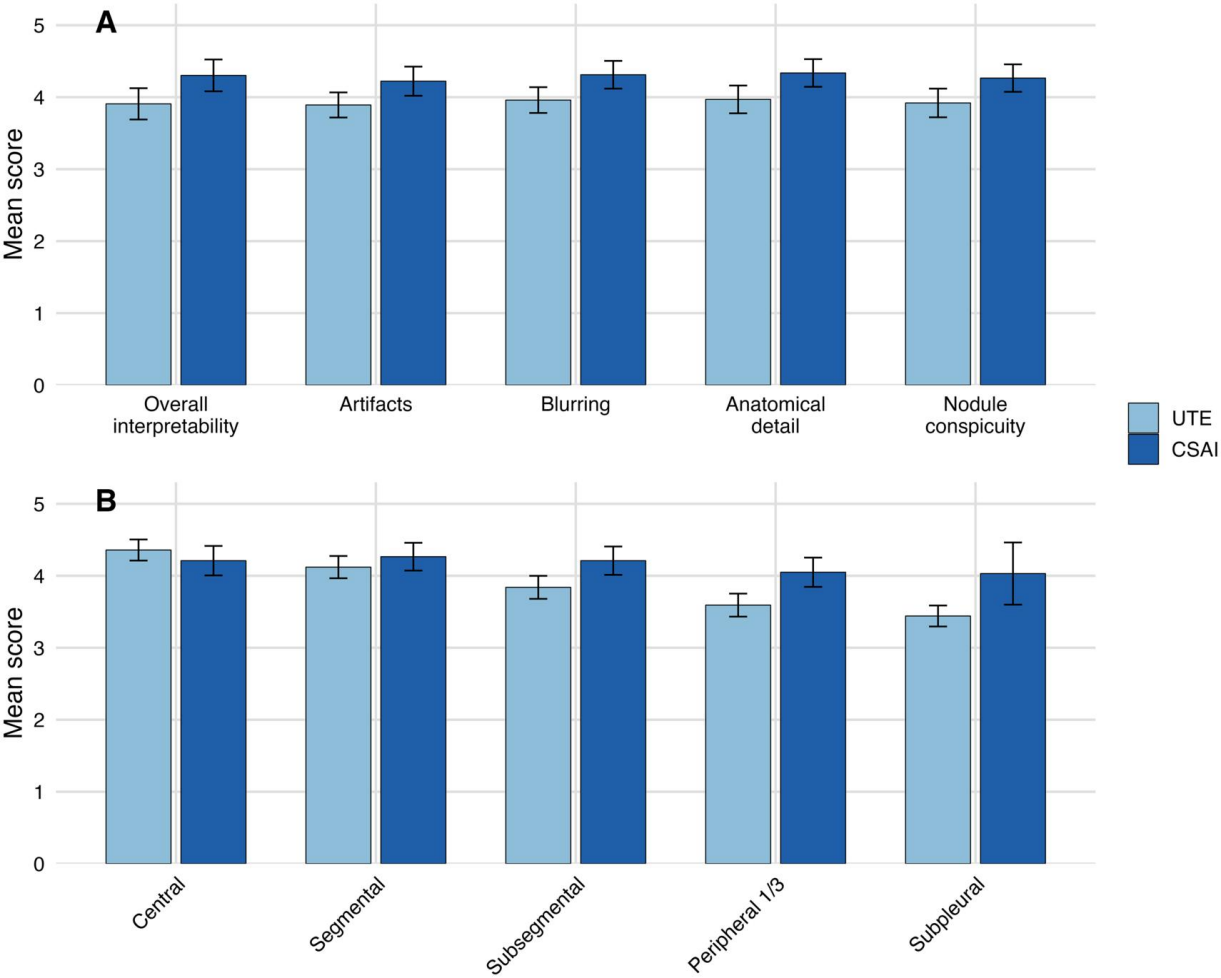
Image quality ratings of CSAI-GRE and UTE MRI. Bars represent mean patient-level image quality scores after averaging ratings across readers for each patient and sequence. Error bars indicate 95% CIs. Image quality was evaluated using a 5-point Likert scale (1 = non-diagnostic, 5 = excellent). (A) Image features. CSAI-GRE demonstrated significantly higher ratings for overall interpretability (*P* = .005), artifacts (*P* = .002), blurring (*P* = .004), anatomical detail (*P* = .005), and nodule conspicuity (*P* = .009) compared with UTE (Wilcoxon signed-rank test). (B) Anatomic region. CSAI-GRE = compressed sensing artificial intelligence-accelerated gradient-echo sequence; UTE = ultrashort echo time sequence.

CSAI-GRE demonstrated significantly higher overall image quality compared with UTE (mean score, 4.30 [95% CI, 4.08-4.52] vs 3.91 [95% CI, 3.69-4.12]; *P* = .005). Domain-specific analysis demonstrated significantly higher ratings for CSAI-GRE across all evaluated domains, including overall interpretability, artifacts, blurring, anatomical detail, and nodule conspicuity (all *P* < .05). Detailed image-quality results are provided in Figure 3 and Table S1.

#### Nodule detection and characterization

At the lesion level, CSAI-GRE detected 282/291 nodules (96.9%) vs 270/291 nodules (92.8%) with UTE (Table 3). Reader-specific detection rates are summarized in Table S2. Mixed-effects analysis accounting for clustered observations demonstrated significantly higher conditional detection odds for CSAI-GRE compared with UTE (OR, 10.2; 95% CI, 2.4-43.3; *P* = .002). In the subgroup of nodules <10 mm, CSAI-GRE detected 153/162 nodules (94.4%), compared with 141/162 nodules (87.0%) for UTE; subgroup mixed-effects analysis demonstrated similarly higher detection odds for CSAI-GRE (OR, 10.1; 95% CI, 2.4-42.8; *P* = .002) (Table S3). No false-positive nodules were detected with either sequence. Three nodules were missed on CSAI-GRE and 7 nodules on UTE. Three small solid nodules were missed by both sequences, whereas 4 additional small solid nodules were missed only on UTE. No part-solid or ground-glass nodules were missed.

**Table 3:** Lesion-level detection performance.

Sequence	Detected	Missed	Detection rate (%)	*P*-value
CSAI-GRE	282/291	9	96.9	.002
UTE	270/291	21	92.8	

Detection rates are reported descriptively as the number of detected nodules divided by the total number evaluated. The lesion-level *P-*value was derived from a generalized linear mixed-effects model with sequence type as fixed effect and random intercepts for patient and reader.

Abbreviations: CSAI-GRE, compressed sensing AI-accelerated 3D gradient-echo sequence; UTE, ultrashort echo time sequence.

**Figure 4:**
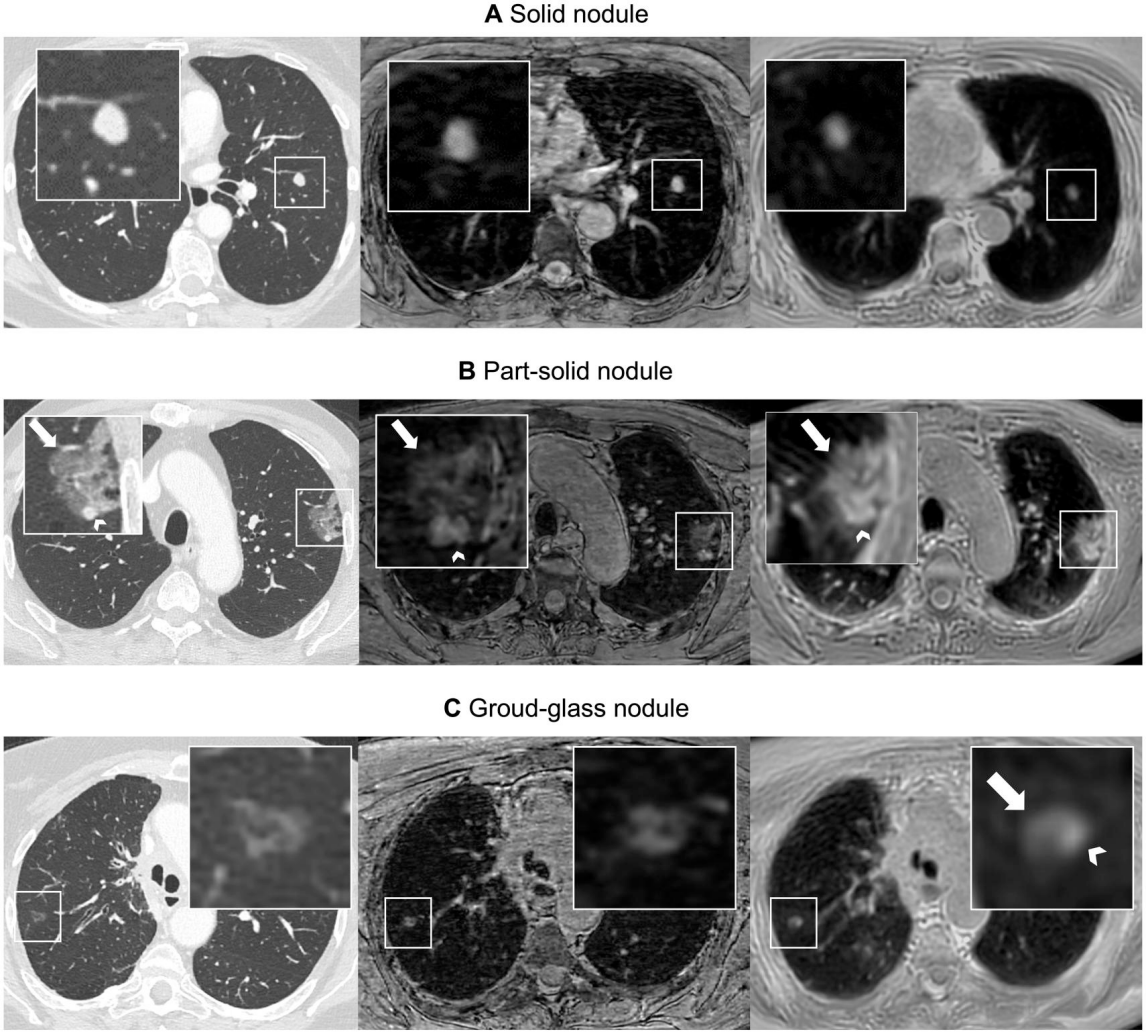
Multimodal examples of pulmonary nodules on CT and MRI. For all panels, images are shown as contrast-enhanced CT, respiratory-gated AI-accelerated 3D gradient-echo MRI with isotropic resolution (CSAI-GRE), and respiratory-gated 3D isotropic UTE MRI using FLORET trajectory from left to right. Insets show magnified views of the lesion. (A) Solid pulmonary nodule in the left upper lobe of an 84-year-old man with prostate cancer, representing a pulmonary metastasis. Mean nodule diameter 13 mm on CT, 12 mm on CSAI-GRE, and 9 mm on UTE. The nodule was classified as solid by all readers on both MRI sequences. Based on its solid attenuation and size between 8 and 15 mm, the nodule was assigned Lung-RADS 4A on CT, CSAI-GRE, and UTE by all readers. (B) Subpleural part-solid pulmonary nodule in the left upper lobe of a 76-year-old man. Mean nodule diameter measured 37 mm on CT, 32 mm on CSAI-GRE, and 38 mm on UTE; corresponding solid-component diameters were 8, 10, and 14 mm. The nodule was classified as part-solid by all readers and assigned Lung-RADS category 4B on all modalities. Histopathology confirmed lepidic-predominant non-small cell lung carcinoma (NSCLC). The thick arrow indicates the ground-glass component, while the arrowhead marks the solid component. (C) Ground-glass pulmonary nodule in the right upper lobe of a 63-year-old woman. Mean nodule diameter was 11 mm on CT, and 10 mm on both CSAI-GRE and UTE. The nodule was classified as ground glass on CT; on CSAI-GRE, 2 readers classified it as ground glass and 1 reader as part-solid, whereas all readers classified it as part-solid on UTE. The nodule was assigned Lung-RADS 2 on CT and CSAI-GRE and Lung-RADS 3 on UTE. Follow-up confirmed an inflammatory nodule. In the UTE inset, the arrowhead marks the component interpreted as solid component, and the thick arrow marks the true ground-glass component.

Inter-reader agreement for nodule attenuation was nearly perfect for both MRI sequences (Fleiss *κ*, CSAI-GRE: 0.89 [95% CI, 0.75-1.00]; UTE: 0.85 [95% CI, 0.72-0.95]). Agreement between MRI and CT for nodule attenuation was substantial for CSAI-GRE (Cohen *κ* range, 0.66-0.82; raw agreement 93.6%-96.8%) and fair to moderate for UTE (Cohen *κ* range, 0.28-0.51; raw agreement 83.3%-87.8%) (Table S4). Exemplary nodules are illustrated in Figure 4.

**Figure 5:**
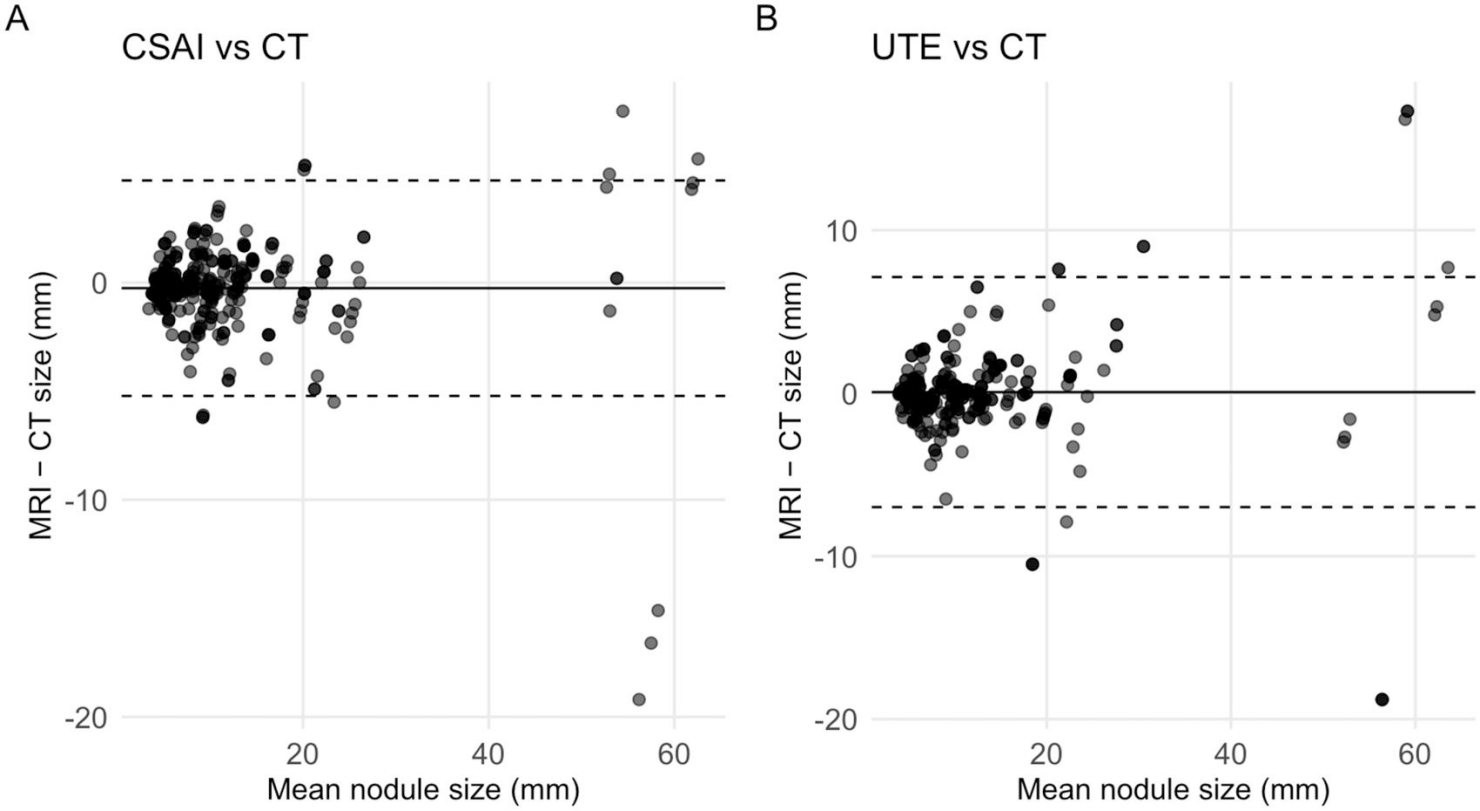
Bland-Altman analysis of pulmonary nodule size measurements on MRI compared with CT. (A) CSAI-GRE vs CT. Mean difference −0.3 mm with 95% limits of agreement −5.1 to 4.6 mm. (B) UTE vs CT. Mean difference 0.1 mm with 95% limits of agreement −7.0 to 7.1 mm. Each dot represents one pulmonary nodule. The solid horizontal line indicates the mean difference (bias) between MRI and CT measurements, while the dashed lines represent the 95% limits of agreement (mean difference ± 1.96 SD). CSAI-GRE = compressed sensing accelerated 3D gradient echo with deep learning reconstruction; UTE = ultrashort echo time sequence.

Mean nodule diameter was assessed on both CSAI-GRE and UTE images. Reader-averaged MRI measurements were compared with CT for nodules detected on each MRI sequence; therefore, the CT reference subset differed slightly between CSAI-GRE and UTE. Mean nodule diameter was 12 ± 11 mm for CSAI-GRE and 13 ± 12 mm for UTE, compared with corresponding CT reference measurements of 13 ± 11 mm for both (Table S5). Measurement agreement between reader-averaged MRI measurements and CT was excellent for both sequences (ICC-CSAI-GRE: 0.975 [95% CI, 0.962-0.983]; ICC-UTE: 0.955 [95% CI, 0.932-0.970]). Compared with CT, CSAI-GRE demonstrated a mean deviation of 0 mm (95% LoA, −5 to 5 mm), whereas UTE showed a mean difference of 0 mm (95% LoA, −7 to 7 mm). Bland-Altman plots confirmed no systematic measurement bias (Figure 5).

#### Lung-RADS category agreement

Inter-reader agreement for Lung-RADS categorization was almost perfect for CSAI-GRE (Fleiss *κ*, 0.86 [95% CI, 0.76-0.94]) and substantial for UTE (Fleiss *κ*, 0.76 [95% CI, 0.65-0.86]). Agreement between MRI- and CT-based Lung-RADS categorization ranged from substantial to almost perfect across readers (Cohen *κ* range = 0.79-0.93 for CSAI-GRE and 0.72-0.94 for UTE; Table S6).

Lung-RADS reclassifications relative to CT occurred in 20/162 (12.3%; 95% CI, 8.1%-18.3%) of CSAI-GRE assessments, including 9 (5.6%; 95% CI, 3.0%-10.2%) critical Lung-RADS reclassifications. For UTE, 33/159 (20.8%; 95% CI, 15.2%-27.7%) of cases showed reclassifications, including 17 (10.7%; 95% CI, 6.8%-16.5%) critical reclassifications. Detailed category distributions are provided in Table 4.

**Table 4:** Lung-RADS category distribution on CT (reference standard), CSAI-GRE, and UTE by reader.

		Reader 1		Reader 2		Reader 3	
Lung-RADS	CT	CSAI-GRE	UTE	CSAI-GRE	UTE	CSAI-GRE	UTE
1	1 (1.9%)	1 (1.9%)	1 (1.9%)	1 (1.9%)	1 (1.9%)	1 (1.9%)	1 (1.9%)
2	15 (27.8%)	16 (29.6%)	14 (26.4%)	14 (25.9%)	15 (28.3%)	14 (25.9%)	14 (26.4%)
3	4 (7.4%)	10 (18.5%)	10 (18.9%)	6 (11.1%)	4 (7.5%)	6 (11.1%)	4 (7.5%)
4A	15 (27.8%)	11 (20.4%)	13 (24.5%)	14 (25.9%)	14 (26.4%)	14 (25.9%)	16 (30.2%)
4B	11 (20.4%)	12 (22.2%)	11 (20.7%)	12 (22.2%)	13 (24.5%)	12 (22.2%)	12 (22.6%)
4X	8 (14.8%)	4 (7.4%)	4 (7.5%)	7 (13.0%)	6 (11.3%)	7 (13.0%)	6 (11.3%)

Values are number of patients with percentages in parentheses, calculated within each column. Column totals = 54, unless otherwise indicated. For UTE, all readers had one case with no detected nodules, and no Lung-RADS category could be assigned; therefore, UTE column totals are *n* = 53 for Reader 1-3. CT denotes the reference standard computed from the corresponding CT examination.

Abbreviations: CSAI-GRE, compressed sensing AI-accelerated 3D gradient-echo sequence; Lung-RADS, Lung Imaging Reporting and Data System (version 2022); UTE, ultrashort echo time sequence.

Most reclassifications involved downgrades from Lung-RADS 4X to lower categories or upgrades from 4A to 4B. For CSAI-GRE, the most frequent critical transitions were 4X to 3 (4/20 reclassifications, 20.0%) and 4A to 4B (3/20, 15.0%). For UTE, the most frequent critical transitions were 4X to 4A (7/33, 21.2%) and 4A to 4B (6/33, 18.2%). Detailed reader-specific reclassification rates and transition patterns are provided in Tables S7a and S7b.

## Discussion

This study compared respiratory-gated 3D UTE and accelerated 3D GRE sequences using CT as the reference for nodule detection and Lung-RADS classification. CSAI-GRE demonstrated comparable agreement with CT for Lung-RADS categorization while achieving slightly higher nodule detection rates and improved subjective image quality compared with UTE.

Detection rates exceeded prior Cartesian GRE studies^7^^,^^22^ and were comparable to radial MRI techniques including UTE and radial GRE.^5^^,^^22^^,^^23^ However, UTE imaging poses technical challenges, especially in attaining isotropic 3D resolution because of intricate reconstruction methods. Standard UTE protocols typically last 4-7 min under respiratory-gated acquisition, depending on the respiratory cycle.^24^ Radial GRE sequences have similar limitations.^25^

Previous comparisons of radial and Cartesian GRE sequences were often limited by differences in slice thickness (between 2 and 5 mm) or acquisition methods,^7^^,^^25–28^ affecting small-nodule visualization. In contrast, both study sequences used isotropic 3D resolution and identical motion management. CSAI-GRE was acquired with 1 mm isotropic voxels, whereas UTE used 1.5 mm isotropic resolution in order to maintain comparable acquisition times with respiratory gating. The high detection rates observed for CSAI-GRE may partly reflect spatial resolution and tissue contrast differences between GRE and UTE sequences. GRE sequences with longer TE (0.83 ms) produce low lung parenchyma signal, potentially increasing lesion conspicuity, whereas UTE sequences capture signals from lung parenchyma by identifying extremely short T2* components,^2^^,^^3^^,^^11^ potentially reducing contrast between nodules and surrounding tissue. Nonetheless, UTE’s lung characterization may benefit comprehensive nodule evaluation beyond initial detection.^29^^,^^30^

CSAI-GRE also demonstrated higher subjective image quality, including anatomical detail and nodule conspicuity. This likely reflects compressed sensing and deep-learning-based reconstruction enabling efficient undersampling while preserving spatial resolution.^31^ Missed nodules mainly occurred in anatomically challenging regions and examinations with moderate image quality. The wide Bland-Altman limits indicate that measurement variability remains clinically relevant, particularly for small pulmonary nodules. Contributing factors include partial-volume effects, MRI intrinsic spatial-resolution limits, residual respiratory motion despite gating, and sequence-dependent tissue contrast and edge definition. Wider UTE limits may reflect its lower isotropic resolution (1.5 mm vs 1.0 mm for CSAI-GRE) and differing parenchymal signal characteristics, which may reduce lesion conspicuity and edge delineation.

Lung-RADS classification was used because it provides a granular evaluation methodology that strongly parallels other nodule evaluation frameworks, such as the recommendations of the Fleischner Society^32^ and the British Thoracic Society.^33^ Lung-RADS categorization demonstrated substantial agreement between MRI and CT for both sequences. However, reclassification relative to CT occurred in 12.3% of CSAI-GRE assessments and 20.8% of UTE assessments, including management-relevant category changes in 5.6% and 10.7% of examinations, respectively. Although these reclassifications occurred in a minority of cases, they underscore the need for further optimization before MRI can be considered for routine clinical decision-making.

One possible application is lung evaluation during MRI examinations performed for other oncologic indications. In patients undergoing abdominal or pelvic MRI for cancer staging or follow-up, simultaneous assessment of the lungs could reduce the need for additional CT imaging. MRI may also be advantageous in pediatric populations or in patients requiring repeated imaging where radiation exposure is a concern. In these contexts, slightly reduced detection performance compared with CT may be clinically acceptable, particularly if MRI can reliably identify clinically significant nodules.

Several limitations should be considered. The cohort included only patients with CT-detected pulmonary nodules, resulting in an enriched population with a higher prevalence of nodules and Lung-RADS ≥4 categories than would be expected in screening populations and reader bias toward nodule detection. This introduced spectrum bias, precluded specificity assessment, and may explain absence of false-positive nodules. In addition, because the study included only CT-confirmed nodules rather than a full diagnostic detection task, accuracy or area under the receiver-operating-characteristic curve (AUC) could not be calculated. Only 3 readers were included, and the estimated reader-level variance in the mixed-effects models approached 0; therefore, reader-level random-effect estimates should be interpreted cautiously. CT served as the reference standard, although histopathologic confirmation was not available for all nodules and benignity was primarily determined by imaging stability on follow-up. The CT-MRI interval of up to 8 days may have contributed to measurement variability, particularly for inflammatory or transient nodules in which subtle interval changes in lesion size or morphology cannot be excluded. Although we did not use breath-hold sequences, respiratory gating improved patient comfort, which is particularly useful for those with respiratory comorbidities. A further limitation is the difference in slice thickness between sequences, which was necessary to maintain similar acquisition times, as UTE would otherwise require a substantially longer acquisition time than CSAI-GRE. This may have introduced bias into the comparison. Additionally, UTE development for pulmonary MRI is still less mature than GRE-based approaches, and future advances in sequence research and technical optimization may affect the relative performance of these techniques. Furthermore, patients with advanced emphysema and severe structural lung disease were excluded, which may limit generalizability to lung cancer screening populations in which parenchymal destruction is common. In addition, measurement variability observed in the Bland-Altman analysis suggests that caution is warranted when applying MRI measurements for longitudinal nodule growth assessment. Finally, the relatively large mean nodule size in the study cohort may overestimate detection performance compared with early screening cohorts where smaller nodules predominate.

Future studies should evaluate CSAI-GRE in larger and more heterogeneous patient populations, including cohorts representative of lung cancer screening and oncologic follow-up settings. In addition, longitudinal studies will be necessary to determine the diagnostic accuracy and reliability of MRI-based measurements for assessing nodule growth over time and to explore advanced quantitative MRI techniques for pulmonary nodule characterization.

In conclusion, CSAI-GRE provides high subjective image quality and reliable pulmonary nodule detection with good agreement with CT for attenuation assessment and Lung-RADS classification. However, the observed rate of critical Lung-RADS reclassifications for both CSAI-GRE and UTE sequences highlights the need for further optimization before MRI-based nodule assessment can be considered for routine clinical decision-making.

## Supplementary Material

umag029_Supplementary_Data

## Data Availability

The data underlying this article will be shared on reasonable request to the corresponding author.
