# Supplementary material for: AI-accelerated 3D gradient echo versus ultrashort echo time MRI for lung nodule detection and measurement
Source: Radiol Adv. 2026 Jul 7;3(4):umag029. doi: 10.1093/radadv/umag029 (PMC13399443; doi:10.1093/radadv/umag029)
Supplement: umag029_Supplementary_Data [file umag029_supplementary_data.zip › Supplement_Data.zip!/Supplementary Material_FINAL.pdf]

Supplementary Material

Title: AI-Accelerated 3D Gradient Echo versus Ultrashort Echo Time MRI for Lung Nodule Detection and Measurement

S1. Detailed CT Acquisition Parameters

Chest CT examinations were performed on a 320–detector row system (IQon – Spectral CT, Philips Healthcare, Best, The Netherlands). All examinations were contrast-enhanced and acquired according to the clinical indication, including oncologic staging/follow-up, infection/inflammatory evaluation, chest pain, and pulmonary embolism assessment.

Acquisition parameters were as follows:

- Tube voltage: 120 kV
- Automated tube current modulation (mean effective tube current: 59 mAs)
- Collimation: 64 × 0.625 mm
- Pitch factor: 1.0
- Acquisition phase: Full inspiration

Reconstruction parameters:

- Reconstruction kernel: Dedicated lung kernel (YA)
- Slice thickness: 1 mm
- Slice interval: 1 mm
- iDose iterative reconstruction level: 6
- Reconstruction plane: Axial (multiplanar reformations available as needed)

Intravenous iodinated contrast material with an iodine concentration of 400 mg iodine/mL was administered in all CT examinations according to institutional clinical protocols. Contrast volume ranged from 50-100 mL, followed by a 30-50 mL saline flush. Injection flow rate ranged from 2-4 mL/s and was adapted to clinical indication and patient body habitus. CT acquisitions included venous-phase chest CT for oncologic staging/follow-up, inflammatory evaluation, or unexplained chest pain, and pulmonary arterial-phase CT for suspected pulmonary embolism.

S2. Detailed MR Acquisition Parameters

MRI examinations were performed on a 3T system (Ingenia Elition X; Philips Healthcare, Best, The Netherlands) using a 16-channel anterior and integrated 12-channel posterior coil.

S2.1 CSAI-GRE Sequence

Respiratory-gated 3D gradient echo imaging was acquired using Cartesian radio frequency spoiled sequence. The acquisition employed non-selective radiofrequency excitation pulse, partial Fourier frequency encoding in readout direction to reduce the effective echo time and

a Cartesian variable-density k-space sampling strategy with a compressed sense-based pseudo random k-space undersampling.

Acceleration factor: 7

Sequence parameters:

- TR: 2.7 ms
- TE: 0.83 ms
- Flip angle: 10°
- Field of view: 320 × 557 × 320 mm^3
- Acquired voxel size: 1 × 1 × 1 mm^3
- Reconstructed voxel size: 0.5 mm isotropic
- Partial Fourier in readout direction (partial echo): 0.65
- Respiratory gating method: Camera based (VitalEye, Philips) triggering
- Estimated acquisition time: ~3:50 min (assuming 3 s respiratory cycle)

Reconstruction was based on the Adaptive-CS-Net (1), a convolutional neural network integrated into an unrolled iterative reconstruction framework. Its architecture builds on a learned iterative shrinkage–thresholding algorithm (ISTA)(2),enabling multiscale sparsification in a problem-aware, data-driven manner. Training was conducted on a dataset of roughly 740,000 anonymized MR images spanning multiple anatomical regions, sequence types, and field strengths (1.5 T and 3 T). The model incorporates physical priors such as coil sensitivity information and background constraints and was optimized to ensure robust generalization across a wide range of clinical scenarios. The reconstruction was implemented using the commercially available SmartSpeed reconstruction platform (Philips Healthcare).

S2.2 UTE Sequence (FLORET)

Respiratory-gated 3D ultrashort echo time imaging was performed using the FLORET (Fermat looped, orthogonally encoded trajectories) trajectory (3-5). The FLORET sequence uses a Fermat-spiral–based sampling pattern that distributes readout points more uniformly than conventional radial UTE trajectories, reducing central k-space oversampling and improving sampling efficiency while preserving SNR for short-T2* signals (6). Each Fermat spiral is mapped onto a cone surface, with successive cones created by incrementally increasing the cone angle and rotating each by the golden angle to ensure uniform coverage. In this study, FLORET employed three hubs with a maximum cone angle of ±45°.

Sequence parameters:

- TR: 9.5 ms
- TE: 0.095 ms
- Flip angle: 8°
- Field of view: 350 × 350 × 350 mm^3
- Voxel size: 1.5 mm isotropic
- Number of hubs: 3
- Maximum cone angle: ±45°
- Respiratory gating method: Camera based (VitalEye, Philips) triggering
- Estimated acquisition time: ~3:30 min (assuming 3 s respiratory cycle)

S3. Definition of Lung-RADS Reclassifications

For each MRI sequence (CSAI-GRE and UTE), Lung-RADS categories assigned on MRI were compared with the reference Lung-RADS category determined from CT.

A reclassification was defined as any difference in Lung-RADS category between MRI and CT for a given nodule.

A critical reclassification was defined as a change in Lung-RADS category that would alter clinical management according to Lung-RADS recommendations, specifically transitions between non-invasive surveillance categories and categories recommending more aggressive diagnostic evaluation.

Accordingly, the following transitions were considered critical:

- Upgrade to a higher-risk category requiring invasive diagnostic evaluation

  (e.g., 4A → 4B, 3 → 4B, 2 → 4B, 1 → 4B, or transitions to 4X)

- Downgrade from a category recommending invasive evaluation to a lower-risk surveillance category

  (e.g., 4B → 4A, 4B → 3, 4X → 4A, 4X → 3)

These transitions were considered clinically relevant because they may change patient management from imaging follow-up to invasive diagnostic procedures or vice versa.

All other category differences were classified as non-critical reclassifications, as they would generally not change the recommended management pathway.

S4. Multidimensional Image Quality Assessment

S4.1 General reading conditions

- All reads performed on standard PACS workstation under routine reading room conditions.
- Readers blinded to clinical data and CT findings.
- Sessions separated by washout as described in main Methods.
- Case order randomized within each session per reader.

S4.2 Patient-level image quality: domains and scales

Patient-level image quality was graded separately for each MRI sequence and each patient.

Global diagnostic threshold

- Scores **≥3** were defined as **diagnostic/interpretable**.
- Scores **1–2** were considered **non-diagnostic or diagnostically unreliable**.

Domain 1 - Overall interpretability (1-5)

1. Non-diagnostic: image cannot be used for nodule assessment

2. Poor: severe limitations; assessment unreliable
3. Acceptable: diagnostic; limitations present but assessment feasible
4. Good: minor limitations only
5. Excellent: fully diagnostic; no relevant limitations

Domain 2 - Artifact severity (1-5)
(artifacts include respiratory motion, streaking, ringing, ghosting, susceptibility-related distortions)

1. Severe artifacts obscuring large regions; non-diagnostic
2. Marked artifacts frequently obscuring lung detail
3. Moderate artifacts present but assessment feasible
4. Minor artifacts without meaningful impact
5. No relevant artifacts

Domain 3 - Blurring (1-5)
(blur due to motion, gating inefficiency, reconstruction smoothing)

1. Severe blurring; margins and vessels not definable
2. Strong blurring; uncertain evaluation of small nodules
3. Moderate blurring; diagnostic but limited detail
4. Mild blurring; does not restrict interpretation
5. Sharp depiction throughout

Domain 4 - Depiction of anatomical detail (1-5)
(ability to delineate bronchovascular structures and lung interfaces)

1. No reliable bronchovascular detail; non-diagnostic
2. Poor: major loss of detail
3. Moderate: segmental/subsegmental detail limited but diagnostic
4. Good: clear segmental/subsegmental depiction
5. Excellent: high-detail depiction including peripheral structures

Domain 5 - Overall nodule conspicuity (patient-level) (1-5)
("overall" impression across nodules/lesions in that patient)

1. Nodules generally not conspicuous
2. Many nodules poorly delineated; confidence low
3. Nodules moderately conspicuous; diagnostic
4. Nodules clearly conspicuous; high confidence
5. Excellent conspicuity; sharp delineation and strong contrast

S4.3 Regional image quality using vascular landmarks (1-5 per region)

Each patient-level scan was also scored for regional image quality based on vascular landmarks:

Regions to score:

1. **Central** (main pulmonary artery level)
2. **Segmental vessels**
3. **Subsegmental vessels**
4. **Peripheral one-third of lung**

5. **Subpleural/pleural region**

Regional score definition (applied to each region)

1. Non-diagnostic region: vessels and parenchyma not evaluable
2. Poor: limited vessel delineation; nodule evaluation unreliable
3. Acceptable: diagnostic; moderate limitations
4. Good: minor limitations
5. Excellent: clear depiction without limitations

Supplementary Tables

Supplementary Table S1: *Image quality ratings*

Domain	CSAI-GRE mean (95% CI)	UTE mean (95% CI)	p-value
**Overall Interpretability**	4.30 (4.08–4.52)	3.91 (3.69–4.12)	.005
**Artifacts**	4.22 (4.02–4.42)	3.89 (3.72–4.07)	.002
**Blurring**	4.31 (4.12–4.50)	3.96 (3.78–4.14)	.004
**Anatomical detail**	4.34 (4.14–4.53)	3.97 (3.78–4.16)	.005
**Nodule conspicuity**	4.27 (4.07–4.46)	3.92 (3.72–4.12)	.009

Footnote: Image quality was assessed using a 5-point Likert scale for multiple image quality domains. Higher scores indicate better image quality. Scores were averaged across readers for each patient before paired statistical comparison between sequences. Comparisons between MRI sequences were performed using paired statistical tests.
CSAI-GRE = compressed sensing artificial intelligence–accelerated gradient-echo sequence; UTE = ultrashort echo time sequence.

Supplementary Table S2: *Reader-specific lesion detection performance*

Reader	Sequence	Lesions detected	Total lesions	Detection rate (%)
Reader 1	CSAI-GRE	94	97	96.9
Reader 1	UTE	90	97	92.8
Reader 2	CSAI-GRE	94	97	96.9
Reader 2	UTE	90	97	92.8
Reader 3	CSAI-GRE	94	97	96.9
Reader 3	UTE	90	97	92.8

Footnote:
Detection rate represents the proportion of pulmonary nodules correctly detected by each reader relative to the total number of nodules evaluated per sequence. Overall detection performance across readers is summarized in Table 3. Statistical comparisons between MRI sequences accounting for clustering by reader and patient were performed using generalized linear mixed-effects models (Supplementary Table S3).
CSAI-GRE = compressed sensing artificial intelligence–accelerated gradient-echo sequence; UTE = ultrashort echo time sequence.

***Supplementary Table S3**: Detection GLMM analysis*

Analysis	OR	95% CI	p-value
**Overall model: CSAI-GRE vs. UTE**	10.2	2.4-43.3	.002
**Nodules <10 mm: CSAI-GRE vs UTE**	10.1	2.4-42.8	.002

Footnote: Detection performance was evaluated using generalized linear mixed-effects models with sequence type as the fixed effect and random intercepts for reader and patient. Because nodules ≥10 mm were detected in all assessments for both sequences, nodule size category was not included as a fixed effect due to complete separation. A separate subgroup model was therefore performed for nodules <10 mm. Odds ratios represent cluster-conditional estimates.

GLMM = generalized linear mixed-effects model; OR = odds ratio;

CI = confidence interval; CSAI-GRE = compressed sensing artificial intelligence–accelerated gradient-echo sequence; UTE = ultrashort echo time sequence.

Supplementary Table S4: *Attenuation agreement*

**Inter-reader agreement**			
**Sequence**	**Fleiss κ**	**95% CI**	
CSAI-GRE	0.89	0.75–1.00	
UTE	0.85	0.72–0.95	
**CT vs. MRI**			
**Sequence**	**Reader**	**κ**	**Raw Agreement (%)**
CSAI-GRE	Reader 1	0.66	93.6
CSAI-GRE	Reader 2	0.73	94.7
CSAI-GRE	Reader 3	0.82	96.8
UTE	Reader 1	0.28	83.3
UTE	Reader 2	0.51	87.8
UTE	Reader 3	0.51	87.8

Footnote: Agreement between MRI and CT for nodule attenuation classification was evaluated using Cohen's kappa coefficient. Kappa values represent agreement beyond chance. Given the predominance of solid nodules in the cohort, κ values should be interpreted in the context of category imbalance; raw agreement percentages are additionally reported to contextualize κ values.

Abbreviations:
κ = Cohen's kappa coefficient; CSAI-GRE = compressed sensing artificial intelligence–accelerated gradient-echo sequence; UTE = ultrashort echo time sequence.

Supplementary Table S5*: Nodule measurement agreement*

Sequence	MRI mean ± SD (mm)	CT mean ± SD (mm)	Mean differences ± SD (mm)	Lower limit of agreement (mm)	Upper limit of agreement (mm)	ICC	95% CI
**CSAI-GRE (n=94)**	12.3 ± 10.7	12.6 ± 11.0	-0.3 ± 2.5	-5.1	4.6	0.975	0.962-0.983
**UTE (n=90)**	12.9 ± 11.5	12.8 ± 11.1	0.1 ± 3.4	-6.7	6.8	0.955	0.932-0.970

Footnote: CT measurements served as the reference standard. Measurement agreement between MRI and CT was assessed using the intraclass correlation coefficient (ICC) and Bland-Altman analysis. Mean difference represents the average MRI–CT measurement difference.

CSAI-GRE = compressed sensing artificial intelligence–accelerated gradient-echo sequence; UTE = ultrashort echo time sequence; ICC = intraclass correlation coefficient; SD = standard deviation.

Supplementary Table S6:*Lung-RADS agreement*

**Inter-reader agreement**		
**Sequence**	**Fleiss κ**	**95% CI**
CSAI-GRE	0.86	0.76–0.94
UTE	0.76	0.65–0.86

**CT vs. MRI**			
**Sequence**	**Reader**	**κ**	**Raw Agreement (%)**
CSAI-GRE	Reader 1	0.79	77.8
CSAI-GRE	Reader 2	0.93	92.6
CSAI-GRE	Reader 3	0.93	92.6
UTE	Reader 1	0.72	63.0
UTE	Reader 2	0.92	86.8
UTE	Reader 3	0.94	88.5

Footnote: Agreement in Lung-RADS classification between MRI sequences and CT was evaluated using Cohen's kappa coefficients for individual readers and Fleiss' kappa for overall inter-reader agreement. Because the cohort was predominantly composed of solid nodules, κ values should be interpreted in the context of category imbalance; raw agreement percentages are additionally reported to contextualize κ values.
CSAI-GRE = compressed sensing artificial intelligence–accelerated gradient-echo sequence; UTE = ultrashort echo time sequence; Lung-RADS = Lung Imaging Reporting and Data System; κ = Cohen's kappa coefficient.

***Supplementary Table S7a**: Lung-RADS reclassification patterns relative to CT (pooled across readers)*

Sequence	Transition	n	% of changes	Critical change
**CSAI-GRE**	4A → 4B	3	15.0%	Yes
**CSAI-GRE**	4X → 3	4	20.0%	Yes
**CSAI-GRE**	4X → 4A	1	5.0%	Yes
**CSAI-GRE**	4B → 2	1	5.0%	Yes
**CSAI-GRE**	2 → 3	2	10.0%	No
**CSAI-GRE**	4A → 2	2	10.0%	No
**CSAI-GRE**	4A → 3	2	10.0%	No
**CSAI-GRE**	2 → 4A	2	10.0%	No
**CSAI-GRE**	4X → 4B	1	5.0%	No
**UTE**	4A → 4B	6	18.2%	Yes
**UTE**	4X → 4A	7	21.2%	Yes
**UTE**	4X → 3	1	3.0%	Yes
**UTE**	4B → 4A	2	6.1%	Yes
**UTE**	4B → 2	1	3.0%	Yes
**UTE**	2 → 3	5	15.2%	No
**UTE**	4A → 3	3	9.1%	No
**UTE**	1 → 2	1	3.0%	No
**UTE**	2 → 4A	1	3.0%	No
**UTE**	3 → 2	2	6.1%	No
**UTE**	4A → 1	1	3.0%	No
**UTE**	4A → 2	2	6.1%	No

Footnote:
CSAI-GRE = compressed sensing AI-accelerated gradient-echo sequence; UTE = ultrashort echo time; Lung-RADS = Lung Imaging Reporting and Data System.
Detailed definitions of a change in Lung-RADS recommendation and a critical change are provided in Supplementary Material S3.
Percentages represent the proportion of each transition among all reclassifications within the respective sequence.

Supplementary Table S7b*: Reader-specific Lung-RADS reclassification rates*

Sequence	Reader	Ratings	Reclassifications	% reclassified	Critical changes	% critical of changes
**CSAI-GRE**	Reader 1	54	12	22.2%	5	41.7%
**CSAI-GRE**	Reader 2	54	4	7.4%	2	50.0%
**CSAI-GRE**	Reader 3	54	4	7.4%	2	50.0%
**UTE**	Reader 1	53	19	35.8%	8	42.1%
**UTE**	Reader 2	53	7	13.2%	4	57.1%
**UTE**	Reader 3	53	7	13.2%	5	71.4%

Footnote:
CSAI-GRE = compressed sensing AI-accelerated gradient-echo sequence; UTE = ultrashort echo time; Lung-RADS = Lung Imaging Reporting and Data System.
Detailed definitions of a change in Lung-RADS recommendation and a critical change are provided in Supplementary Material S3.
Percentages for reclassifications were calculated relative to the number of Lung-RADS assessments per reader (54 for CSAI-GRE and 53 for UTE). Percentages for critical changes represent the proportion of critical reclassifications among all reclassifications for the respective reader.

Supplementary References

1. Pezzotti N, Yousefi S, Elmahdy MS, Gemert JHFV, Schuelke C, Doneva M, Nielsen T, Kastryulin S, Lelieveldt BPF, Osch MJPV, Weerdt ED, Staring M. An Adaptive Intelligence Algorithm for Undersampled Knee MRI Reconstruction. IEEE Access 2020;8:204825-204838. doi: 10.1109/ACCESS.2020.3034287
2. Zhang J, Ghanem B. ISTA-Net: Interpretable Optimization-Inspired Deep Network for Image Compressive Sensing. IEEE/CVF Conference on Computer Vision and Pattern Recognition 2018:1828-1837. doi: 10.1109/CVPR.2018.00196
3. Willmering MM, Robison RK, Wang H, Pipe JG, Woods JC. Implementation of the FLORET UTE sequence for lung imaging. Magn Reson Med 2019;82(3):1091-1100. doi: 10.1002/mrm.27800
4. Ziegelmayer S, Van AT, Weiss K, Marka AW, Lemke T, Scheuerer F, Huber T, Sauter A, Robison R, Gawlitza J, Makowski MR, Karampinos DC, Graf M. Leveraging Phase Information of 3D Isotropic Ultrashort Echo Time Pulmonary MRI for the Detection of Thoracic Lymphadenopathy: Toward an All-in-One Scan Solution. Investigative Radiology 2024:10.1097/RLI.0000000000001135. doi: 10.1097/rli.0000000000001135
5. Su KH, Friel HT, Kuo JW, Al Helo R, Baydoun A, Stehning C, Crisan AN, Traughber MS, Devaraj A, Jordan DW, Qian P, Leisser A, Ellis RJ, Herrmann KA, Avril N, Traughber BJ, Muzic RF, Jr. UTE-mDixon-based thorax synthetic CT generation. Med Phys 2019;46(8):3520-3531. doi: 10.1002/mp.13574
6. Robison RK, Anderson Iii AG, Pipe JG. Three-dimensional ultrashort echo-time imaging using a FLORET trajectory. Magnetic Resonance in Medicine 2017;78(3):1038-1049. doi: https://doi.org/10.1002/mrm.26500
